# ROR2 deficit may induce the tetralogy of Fallot via down‐regulating of β‐catenin/SOX3/HSPA6 in vitro and in vivo

**DOI:** 10.1111/jcmm.17969

**Published:** 2023-09-25

**Authors:** Rui Guo, Yiran Zhou Guo, Qing Zhou, Guoju Li, Zhanghui Du, Yefei Shi, Quansheng Xing

**Affiliations:** ^1^ Qingdao University Qingdao China; ^2^ Xuzhou Medical University Xuzhou China; ^3^ The Affiliated Hospital of Xuzhou Medical University Xuzhou China; ^4^ An Affiliated Hospital of Women and Children Qingdao University Qingdao China

**Keywords:** congenital heart disease, heat shock protein A6 (HSPA6), receptor tyrosine kinase‐like orphan receptor (ROR2), SRY‐Box Transcription Factor 3 (SOX3), TOF, β‐Catenin

## Abstract

Tetralogy of Fallot (TOF) is the highly conventional appearance of cyanotic congenital heart disease. Our study aimed to assess the involvement of receptor tyrosine kinase‐like orphan receptor 2 (ROR2) in TOF and elucidate the specific mechanism. Upon investigation of human tissue samples, we observed a decrease in *ROR2* expression in TOF patients compared to healthy control individuals. Transcriptome analysis revealed diminished *ROR2* expression in TOF pathological samples relative to normal tissues. Of the 2246 genes that exhibited altered expression, 886 were upregulated, while 1360 were down‐regulated. KEGG analysis and GO analysis of the differentially expressed genes indicated that these genes were significantly enriched in the Wnt signalling pathway, apoptosis and cardiac development function. Importantly, *ROR2* was the only gene shared among the three pathways. Furthermore, interference with ROR2 promotes apoptosis and curtails cell proliferation in vitro. The knockdown of the *ROR2* gene in AC16 cells resulted in a significant decrease in Edu‐positive cells. Flow cytometry studies indicated an increase in the percentage of cells in the S phase. In contrast, the G2/M cell cycle transition was blocked in the *ROR2*‐knockdown group, leading to a significant increase in apoptosis. Moreover, the CCK‐8 cell viability assay demonstrated a reduced proliferation in the *ROR2*‐knockdown group. Furthermore, both in vivo and in vitro data indicated that the expression of HSPA6 (Recombinant Heat Shock 70 kDa Protein6), an essential gene enriched in cardiac tissue and associated with apoptosis, was down‐regulated following *ROR2* knockdown mediated by the β‐catenin/SOX3 signalling pathway. In conclusion, low expression of ROR2 plays a crucial role in the occurrence and development of TOF, which may be related to the downregulation of HSPA6 through the β‐catenin/SOX3 signalling pathway.

## 1 INTRODUCTION

Tetralogy of Fallot (TOF) is one of congenital heart disease's most common congenital disabilities.^1^ Four classical features characterize the TOF, including ventricle septal defect, pulmonary stenosis, aorta overriding and right ventricular hypertrophy.^2^ About 3.5% of new born congenital heart disease patients have TOF, and men and women are equally affected.^3^ With current surgical advancements and management, TOF patients can survive till their 30s and beyond.^4^ Genetic alteration, environmental factors and interactions may disrupt heart development, resulting in TOF.^5^

Receptor tyrosine kinases (RTK) frequently play critical roles in numerous cell‐to‐cell interactions and developmental morphogenesis types.^6^ These receptors interact with soluble ligands or membrane‐associated ligands expressed on neighbouring cells, initiating intracellular signalling cascades.^7^ Ultimately, this leads to cellular responses, including cell migration, proliferation and apoptosis. Recent studies have identified receptor tyrosine kinase‐like orphan receptor (ROR2) as a novel therapeutic and diagnostic target.^7^, ^8^ ROR2 is a member of the RTK superfamily of surface receptors and may play an integral role in the normal development of cardiac tissue.^9^, ^10^, ^11^, ^12^, ^13^ Besides, studies have shown that ROR2 is required for embryonic cardiac development, including septal formation.^9^, ^14^ ROR and Frizzled receptors also mediate the Wnt/β‐catenin‐independent signalling.^15^ Over the decade, the SRY‐Box Transcription Factor (SOX) family of transcription factors was described as playing an essential role in embryonic development.^16^, ^17^ There are more than 20 SOX proteins encoded in the vertebrate genome. Besides, previous studies demonstrated that SOX3 interacts with β‐catenin and regulates the Wnt/β‐catenin pathway target gene.^18^, ^19^

A previous study demonstrated the heat shock protein (HSP) family generally regulate protein folding under synthesis or stress condition, thereby preventing excessive protein degeneration and apoptosis.^20^, ^21^ Although HSPA6 is vastly conservative, very little information is available about it. In addition, HSPA6 (HSP70) is speculated to be overexpressed in various cancer cell lines, tumours and cardiac tissue.^22^, ^23^, ^24^ Given the complexity of heart development, it is hardly surprising during cardiogenesis, TOF development is affected by several transcription factors or molecules. Therefore, this study sought to evaluate the synergistic effect of the ROR2 pathway with β‐catenin and SOX3 in TOF in vivo and in vitro. To identify whether the ROR2 regulates the HSPA6 through the β‐catenin/SOX3 in the development of TOF.

## 2 MATERIALS AND METHODS

### 2.1 Human tissue samples

In six patients with TOF, 3–5 g of tissue was taken between the origin of the pulmonary artery and the right ventricle during the correction of tetralogy of Fallot; the heart specimen was taken from a site that had barely been touched by hypertrophic tissue. The control heart tissue was taken from six organ donors without congenital heart disease, and the tissue was located in the same location as the heart tissue of the TOF patients. The tissues were rapidly frozen in liquid nitrogen and then stored in a −80°C refrigerator. Three normal myocardial tissues and three TOF myocardial tissues were used for transcriptome sequencing, and the other three were used to validate transcriptome results and follow‐up experiments. Query Table S3 for details.

### 2.2 Animal model preparation

Sprague–Dawley rats (1 week old) were obtained from the Experimental Animal Center of Xuzhou Medical University. At Week 1, SD rats were intraperitoneally injected with 4 μL of AAV9‐ROR2‐RNAi (GeneChem, 105516‐1), and the control group was injected with 4 μL of AAV9CON534 (GeneChem). All animals were housed in rooms with a 12‐h diurnal cycle and had AD libitum access to food and water. The rats were followed up for 3–5 days to observe the changes in mental status, diet and physical fitness. All animals were treated in accordance with prescribed Good animal practices. The rats were euthanized 5 weeks after the virus injection. All operations were performed under the regulations of the Animal Care Center Use Committee of Xuzhou Medical University (202006w010).

### 2.3 Cell transfection

The human cardiomyocyte derivative AC16 cell line (Zhongke quality inspection Biotechnology CO, Ltd.) was divided into two groups to transfect with small interference RNA, including the control group (sense: 5′‐UUCUCCGAACGUGUCACGUTT‐3′, antisense: 5′‐ACGUGACACGUUCGGAGATT‐3′), and targeting ROR2 (sense: 5′‐GAGCCAGUAAACAAUAUCATT‐3′, antisense: 5′‐UGAUAUUGUUUACUGGCUCTT‐3′). The siRNA was acquired from Genepharma. Each experiment was performed three times.

### 2.4 CCK8 kit analysis

CCK8, the full name of the Cell Counting Kit‐8 reagent, can be used for simple and accurate cell proliferation and toxicity analysis. Wild‐type AC16 cells and *ROR2* knockdown AC16 cells were cultured in cell culture. After the cell fusion reached 90%, the AC16 cells and the AC16 that knocked down the *ROR2* gene were digested by pancreatic enzyme, respectively, and the plates were grown from 5000 cells per well to 96‐well plates. After incubation for 0, 12, 24, 36, 48 and 72 h, 10 μL CCK8 solution (Yeasen) was added to each well, and the 96‐well plates were continuously incubated for 2 h at 37°C in a 2% CO_2_ incubator. A microplate reader measured the absorbance value of each well solution, and AC16 cell viability was measured by 450 nm optical filter (BioTek).

### 2.5 RT‐qPCR

The human tissue (normal and TOF) and Rat tissue (Control and ROR2‐KD) or human cardiomyocytes AC16 homogenate (100 μL) were placed in the reaction tube. Total RNA was extracted from tissues and cell lines with the TRIzol reagent (Solarbio) according to the manufacturer's protocol. cDNA was synthesized with the PrimeScript RT Reagent Kit (Roche). Real‐time quantitative PCR (qRT‐PCR) analyses were performed with the SYBR Green Premix Ex Taq (Takara). Primers used for PCR were synthesized by Genepharma. The sequence is as follows: Glyceraldehyde‐3‐phosphate dehydrogenase (GAPDH) Sense: 5′‐UGACCUCAACUACAUGGUUTT‐3′ and antisense: 5′‐AACCAUGUAGUUGAGGUCATT‐3′, ROR2 sense: 5′‐ATGGTTCACGACTGCGAATCC‐3′ and antisense: 5′‐ATGGTTCACGACTGCGAATCC‐3′. Each experiment was repeated three times.

### 2.6 RNA sequencing and bioinformation analysis

Total RNA was extracted in the manner described in the previous section. The RNA easy micro kit (QIAGEN, GmBH) and the RNase‐Free DNase Set (QIAGEN, GmBH) were purified and sequenced. To confirm the insert size and determine the mole concentration, purified libraries were measured using a Qubit®2.0 fluorometer (Invitrogen) and validated using an Agilent 2100 bioanalyzer (Agilent Technologies). Clusters were generated by cBot using a diluted library of 10 pM and sequenced on an Illumina HiSeq 2500 platform (Illumina) using a two‐terminal sequencing model (Berry Genomics). EdgeR was used to perform the differentially expressed gene (DEG) analysis (*p* ≤ value 0.05, fold change ≥ 2). R3.6.3 software, including the ggplot2 and cluster profile packages, was used to perform the heat map, volcano plot, gene ontology (GO) analysis and visualization.

### 2.7 Flow cytometry

After 48 h transfection, cells were trypsinized (0.25%) without EDTA, collected in a flow tube and centrifuged to remove the supernatant. Cell pellets were centrifuged to remove the supernatant. Annexin‐V/fluorescein isothiocyanate (FITC) staining buffer (Shuojia Biotech) was prepared according to the manufacturer's instructions. Annexin‐v‐fits, PI and HEPES buffer from the kit were mixed in a 1:2:50 ratio. 1 × 10^6^ cells per 100 μL Annexin‐V/propidium iodide (PI) staining buffer were resuspended and incubated with 1 mL HEPES buffer for 15 min. Apoptosis detection was performed by Flow cytometry (BD Accouri TM C6 Plus, BD Biosciences). The excitation wavelength was 525 nm, and the maximum absorbance of FITC was 488 nm. The maximum absorption wavelength of the PI‐DNA complex was 535 nm, and the maximum emission wavelength was 615 nm. Each experiment was repeated three times.

### 2.8 Western blot

Proteins were extracted from the corresponding tissues or cells using radioimmunoprecipitation assay lysis buffer supplemented with phenylmethylsulfonyl fluoride (R001, Solarbio) precooled at 4°C. The protein concentration in each sample was determined using a Bicinchoninic acid (BCA) kit (Yeasen). Equal amounts of proteins in the samples were separated by sodium dodecyl sulfide‐polyacrylamide gel electrophoresis (SDS‐PAGE) and transferred to a polyvinylidene difluoride (PVDF) membrane. Membranes were blocked with 5% BSA for 1 h at room temperature and incubated with anti‐rabbit primary antibody overnight at 4°C. The primary antibodies used were anti‐ROR2 antibody (1:1000, Abcam, Rats ab190145), anti‐SOX3 antibody (1:1000, GeneTex, rabbit, GTX129235), and anti‐β‐catenin antibody (1:800, Wanleibio, Rats AB190145). rabbit (WL0962a), HSPA6 antibody (1:1000, Proteintech, rabbit, 13616‐1‐AP) and β‐tubulin antibody (1:1000, Abcam, rabbit, ab179511). After incubation with the primary antibody, the cells were washed three times with TBST and incubated with a fluorescent secondary antibody for 2 h at room temperature. The membrane was then washed three times with TBST, and the resulting image was obtained using the Odyssey CLX instrument detection. The results were analysed using ImageJ software. The relative protein content was expressed as the grey value of the corresponding protein band and the grey value of Tubulin. Each experiment was repeated three times.

### 2.9 IHC staining

The myocardial tissue of SD rats and humans was obtained, respectively. Heart tissue was fixed in 4% paraformaldehyde for 24 h, dehydrated in gradient ethanol, embedded in paraffin and sectioned to a thickness of 4 μm. The sections were dewaxed with xylene, rehydrated with upgraded ethanol and washed three times with distilled water. After antigen repair, sections were boiled in citrate buffer for 11 min. After cooling, sections were washed in Tris buffer and incubated in bovine serum albumin (BSA) for 10 min. After antigen retrieval, the sections were incubated in the primary antibody (Rat monoclonal antibody ROR2 antibody (1:500, Abcam, human, ab218105) for 1 h at room temperature. Sections were washed in Tris buffer (pH: 7.4) and then incubated in goat anti‐rats secondary antibody (1:2000, Abcam, ab6789) for 1 h. The samples were incubated with DAB for 10 min, rinsed with Tris buffer (pH: 7.4), counterstained with haematoxylin, rinsed with tap water, dehydrated with graded alcohol, cleared with xylene and covered with the cover sheet. The density of ROR2 was observed in coronal sections with ×400 magnification using a microscope (OlympusAX70). The results were analysed using Image J software, the corresponding OD values were read, and statistical analysis was performed. The specific operation is as follows: (1) Open the picture, Image, type, 8‐bit to convert the picture to a grayscale picture. Convert 8‐bit grayscale picture to OD value: Analyse, Calibrate, select Uncalibrate OD to calibrate; (2) Select the parameters to be measured: Select Set Measurements under ImageJ software Analyse to set the measurement parameters, select Integrated density, Area, Limit to threshold (Image‐> Adjust‐> Threshold adjusts the threshold, red represents selected, select Limit to threshold to calculate the area selected by red only), click OK; (3) Analyse, Measure (Ctrl+M) and get the OD value.

### 2.10 Immunofluorescence (IF)

The SD rats were euthanized 4 weeks after the AAV9 injection. Rats (*n* = 6) were sedated with a high dose of pentobarbital sodium (160 mg/kg) and transcardially reperfused with 0.01 M of PBS and fixed with 4% paraformaldehyde (PFA) in 0.01 M of PBS. The heart was taken and left overnight in 4% PFA, 20% and 30% sucrose solutions. These tissues were fixed in an embedding medium (OTC) and cut into 20 μm sections using a microtome (Leica, CM1950). All sections were preserved in cryoprotectant until further use. The sections were rinsed in PBS (0.01 M × 5 min followed by two times), blocked with 10% normal goat serum (1% BSA, 0.3% Triton X in PBS) and incubated overnight at 4°C with specific primary antibodies: anti‐ROR2 (1:100, Santacruz, Rats, sc‐374174), SOX3 antibody (1:400, GeneTex, rabbit, GTX129235), β‐catenin antibody (1:400, Wanleibio, rabbit, WL0962a), HSPA6 antibody (1:400, proteintech, rabbit, 13616‐1‐AP) and β‐tubulin (1:400, Abcam, rabbit, ab179511). Then, the sections were rinsed in PBS (0.01 M × 5 min followed by two changes) and incubated with a conjugated secondary antibody (Alexa Fluor 488 or Alexa Fluor 594, Abcam or Alexa fluor 777, Abcam). The sections were mounted onto slides and cover‐slipped with Vectashield (Vector Laboratories, Cat. No.: H‐1000). IF results were acquired on a confocal fluorescence microscope Lecia STELLARIS 5.

### 2.11 Construction of AC16 cells overexpressing the *ROR2* gene

When AC16 cells reached 75% confluence, the overexpression plasmid *ROR2*‐pcDNA3.1 was constructed by cloning the full‐length complementary CDS sequence of human ROR2 by Youbao Bio by adding overexpression plasmid (Unibio) and liposome transfection reagent lLIPO3000 (Thermo). The solution was replaced after 6 h, and the *ROR2* overexpression efficiency was verified after 48 h.

### 2.12 Statistical analysis

Data were expressed as mean ± standard deviation (SD) and analysed by GraphPad. *p* < 0.05 was considered statistically significant. Data with normal distribution and homogeneity of variance were compared between the two groups by unpaired *t*‐test. One‐way analysis of variance (anova) was used to compare data between different groups, and the post hoc Tukey test was used. Repeated measures anova with post hoc Tukey test was used to compare data at multiple time points between different groups.

## 3 RESULTS

### 3.1 ROR2 was a low expression in Tetralogy of Fallot patients

**FIGURE 1:**
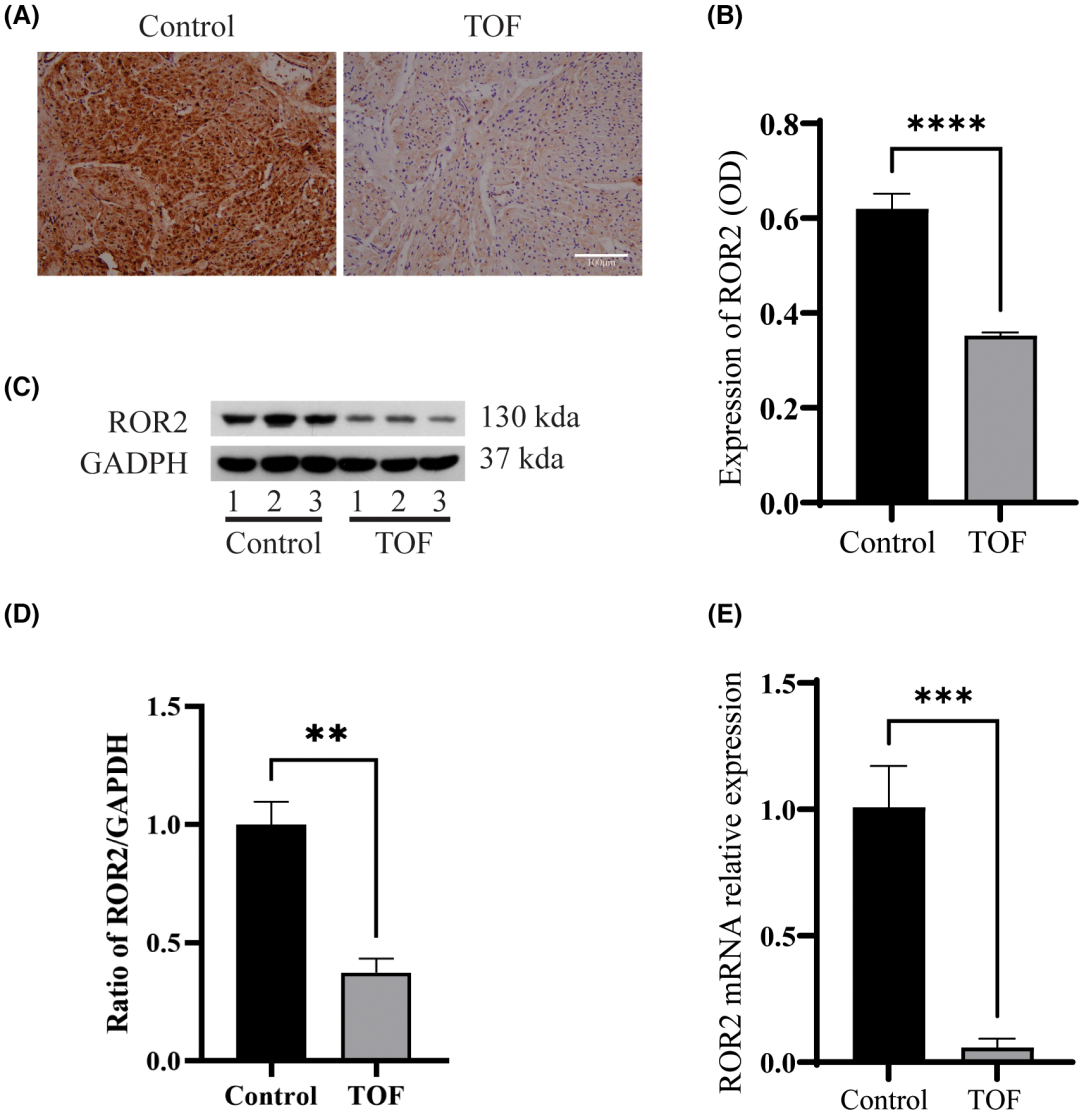
ROR2 was down‐regulated in the Tetralogy of Fallot patients. (A) Representative image of the IHC of the right ventricle of the control and TOF patients, Bar = 100 μm. (B) Representing the collaboration analysis of the IHC image. (C) Representing western blot image of the right ventricle tissue. (D) Representing the quantitative analysis of the ROR2. (E) Representing the mRNA expression of the *ROR2* in right ventricle tissue samples.

To detect whether the ROR2 expression is altered in Tetralogy of Fallot patients: The expression of ROR2 was detected by the immunohistochemistry (IHC), and the analysis revealed that the expression of ROR2 was significantly decreased in the TOF patients compared to the control group (Figure 1A,B, *p* < 0.0001). Furthermore, the protein level was analysed by western blotting (western blot), and the quantification of western blot results showed that the ROR2 protein level was significantly higher in the control compared to the TOF (Figure 1C,D, *p* < 0.001). Similarly, mRNA analysis reveals a low level of ROR2 expression in the TOF compared to the control group, which was consistent with western blot results (Figure 1E, *p* < 0.001). All these results suggest that ROR2 is down‐regulated in TOF.

### 3.2 RNA‐seq in the human heart tissues

**FIGURE 2:**
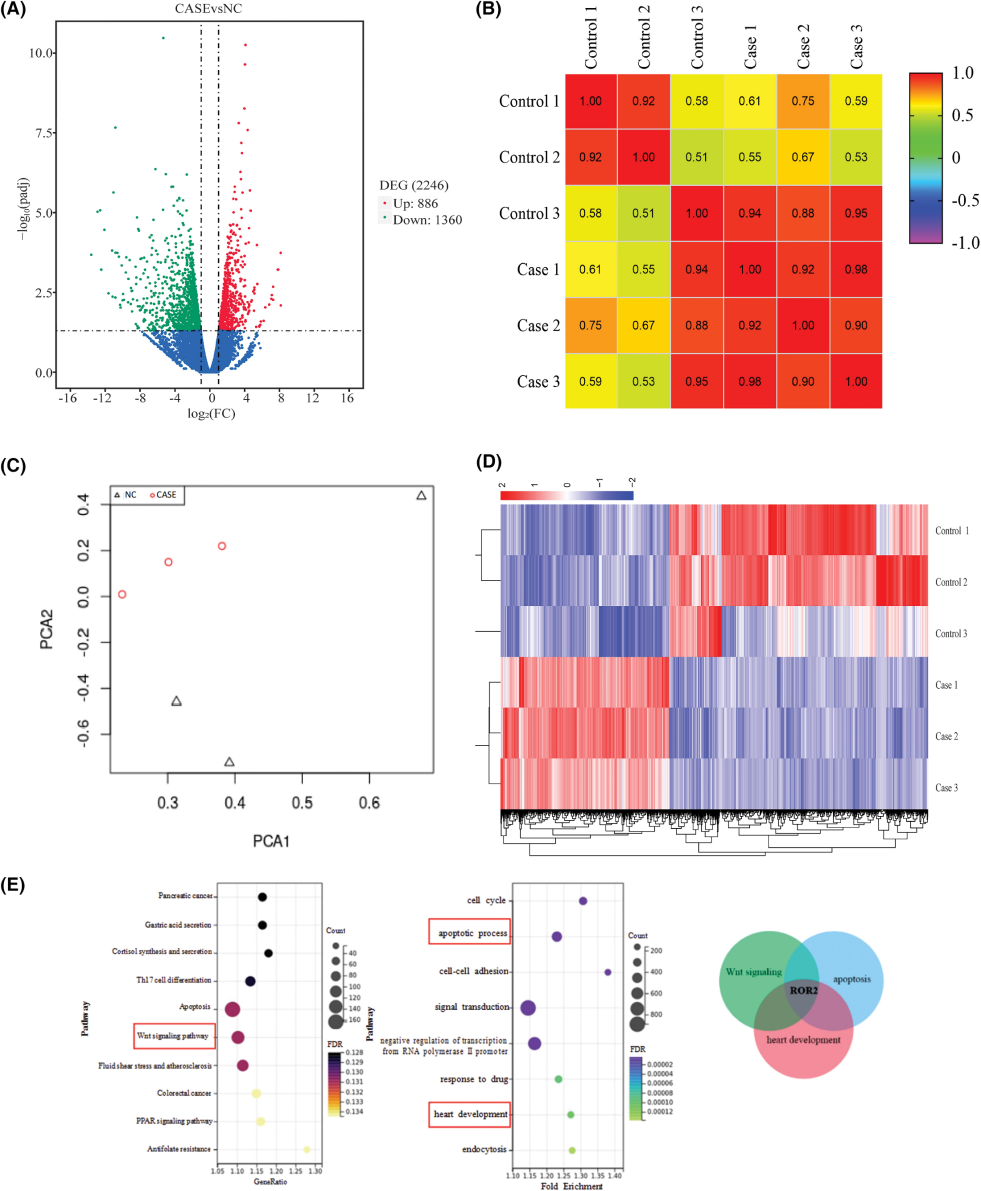
Whole genomic expression of the human right ventricle tissues. (A) A volcano plot analysis represents the genomic expression's magnitude effect on TOF. (B) Represents the principal component analyses of the gene expression of the control group and TOF patients in the heart. (C) To evaluate the difference between groups and the reproducibility of samples within groups, red represents the TOF group, and blue is the normal group. (D) Represents the heat map shows high expression in red and low expression in blue. (E) KEGG analysis and GO analysis of differentially expressed genes were performed. Wnt signalling pathway, apoptosis Venn diagram was used to compare the common genes among the three groups.

To study the potential interaction between the expression of genes and TOF, the transcriptome sequencing analysis was performed on the heart tissue samples of two groups, including control (healthy individuals) and TOF patients. A volcano plot analysis was performed to show the DEGs between the two samples. DEGs were screened using the following criteria: |log2(fold change)| ≥ 1 and *p* < 0.05, and 886 upregulated and 1360 down‐regulated genes (Table S1) are shown in a volcano plot (Figure 2A). Principal component analysis was used to investigate the potential similarity of cardiac gene expression between control and TOF patients. Analysis indicated the separate clustering of both groups, suggesting members of gene expression in both groups are highly correlated and with limited variation between them (Figure 2B). The correlation of gene expression between samples is an important index to test the reliability of experiments and the rationality of sample selection. The closer the correlation coefficient is to 1, the higher the similarity of expression patterns between samples. According to the expression values of all genes in each sample, the correlation coefficients of samples within and between groups were calculated, and the heat map correlation matrix was drawn to visually display the differences between groups and the repetition of samples within groups (Figure 2C). This conclusion is further supported by the hierarchical clustering of samples and genes in the heat map, which indicates that genetic alterations have a more pronounced effect on TOF. The heat map shows high expression in red and low expression in blue (Figure 2D). KEGG analysis and GO analysis of differentially expressed genes showed that the differentially expressed genes were significantly enriched in the Wnt signalling pathway, apoptosis process and development of the heart, and *ROR2* was the only common gene in the three samples. Therefore, in this study, we focused on the potential roles of the *ROR2* gene in the TOF (Figure 2E).

### 3.3 In vitro, interference with ROR2 promotes apoptosis and curtails cell proliferation

**FIGURE 3:**
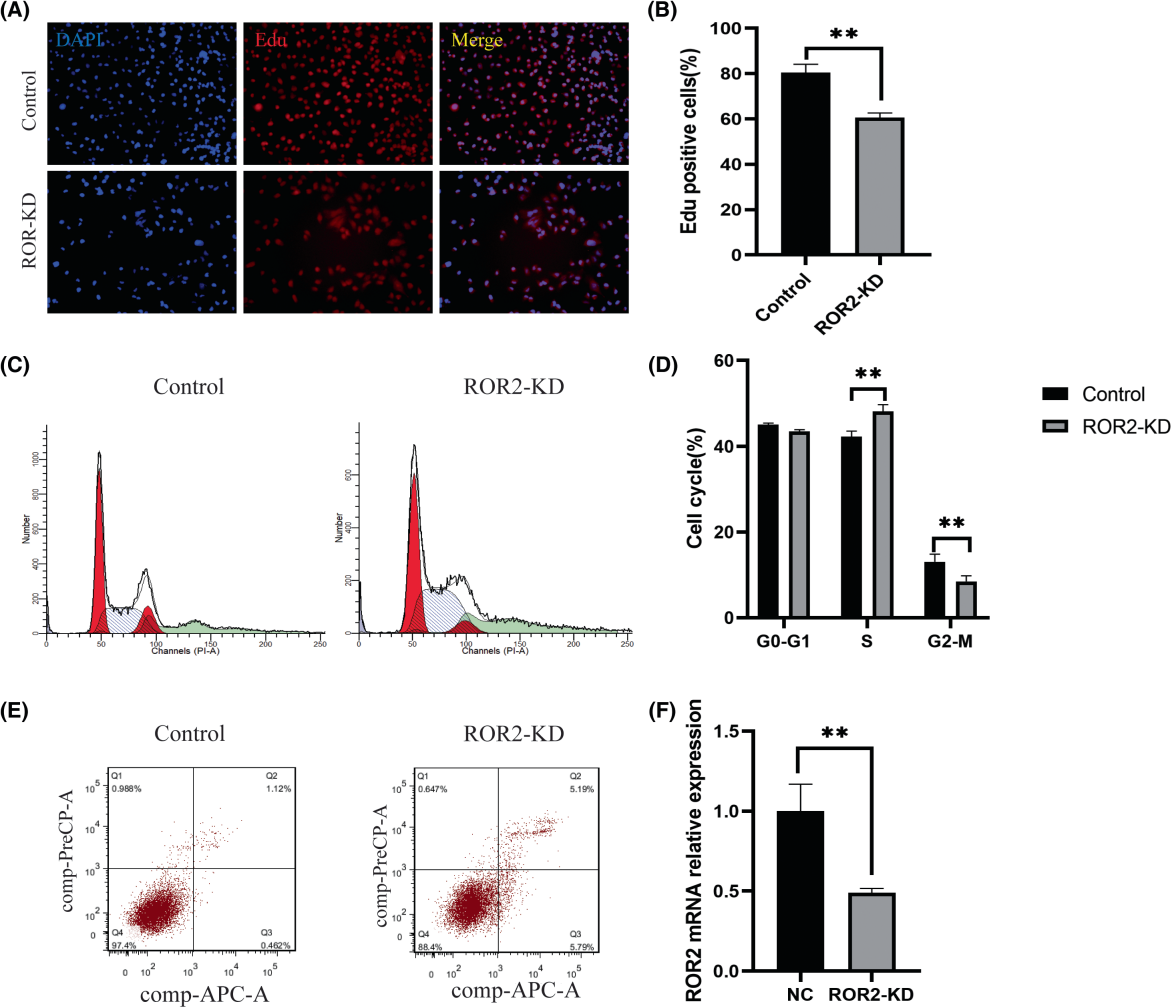
In vitro, depletion of ROR2 induces apoptosis and inhibits proliferation. (A) Representing the Edu expression in the AC16 cell line of the transfected and control group. (B) representing analysis of the Edu‐positive cells. (C) Represents the flow cytometry images of the AC16 cell line, indicating the difference between the cell cycle of both groups. (D) Represents the quantitative analysis of the cell cycle of the AC16 cell line. (E) Represents the flow cytometry that shows the apoptosis of ROR2‐KD and control cells after FITC Annexin‐V and PI staining (F). Represents the mRNA expression of the ROR2 in the AC16 cell line samples.

To further elucidate the impact of ROR2 depletion on cell proliferation and apoptosis, we employed the AC16 cell line. The cells were transfected with small interfering RNA (siRNA) to knockdown the *ROR2* (ROR2‐KD) in an appropriate environment. The normal cells showed a high proportion of the Edu‐positive cells compared to the *ROR2*‐KD (Figure 3A,B, *p* < 0.05). This indicated that after knocking down ROR2, the growth rate of cells gradually decreased. Furthermore, we observe the migration activity of cells at 24 and 48 h after cutting the cell cluster. The analysis reveals that the cell migration activity was significantly decreased in the *ROR2*‐KD cell compared to the control group (Figure S1A,B, *p* < 0.001). Moreover, *ROR2*‐KD and control group cell lines were performed in the transwell chambers with non‐Matrigel or Matrigel, and the cell was stained with crystal violet. The results indicated a significant reduction in the invasion and migration capability of the *ROR2*‐KD group (Figure S1C,D, *p* < 0.05). A flow cytometry study was conducted to determine whether the growth inhibitory effects of *ROR2*‐KD were cell cycle‐dependent. The number of arrests in each cycle phase (including the G1, G2 and S phases) was measured. The analysis indicated that the percentage of the cell cycle in the S phase was elevated in the ROR2‐KD group; nevertheless, the G2/M cell cycle stagnated compared to the control group (Figure 3C,D, *p* < 0.001). The flow cytometry showed that ROR2‐KD cells' apoptosis significantly increased after FITC Annexin‐V and PI staining (Figure 3E). In addition, cell proliferation was examined by the CCK‐8 cell viability assay. The results showed a decreased proliferation in the ROR2‐KD group at 12, 24, 36, 48 and 72 h (Figure S1E, *p* < 0.001). Moreover, the RT‐qPCR results indicated that the expression of the ROR2 was decreased in the KD group (Figure 3F). All the results suggest that ROR2 is essential in regulating cell proliferation and apoptosis.

### 3.4 RNA‐seq in *ROR2* knockdown AC16 cells

**FIGURE 4:**
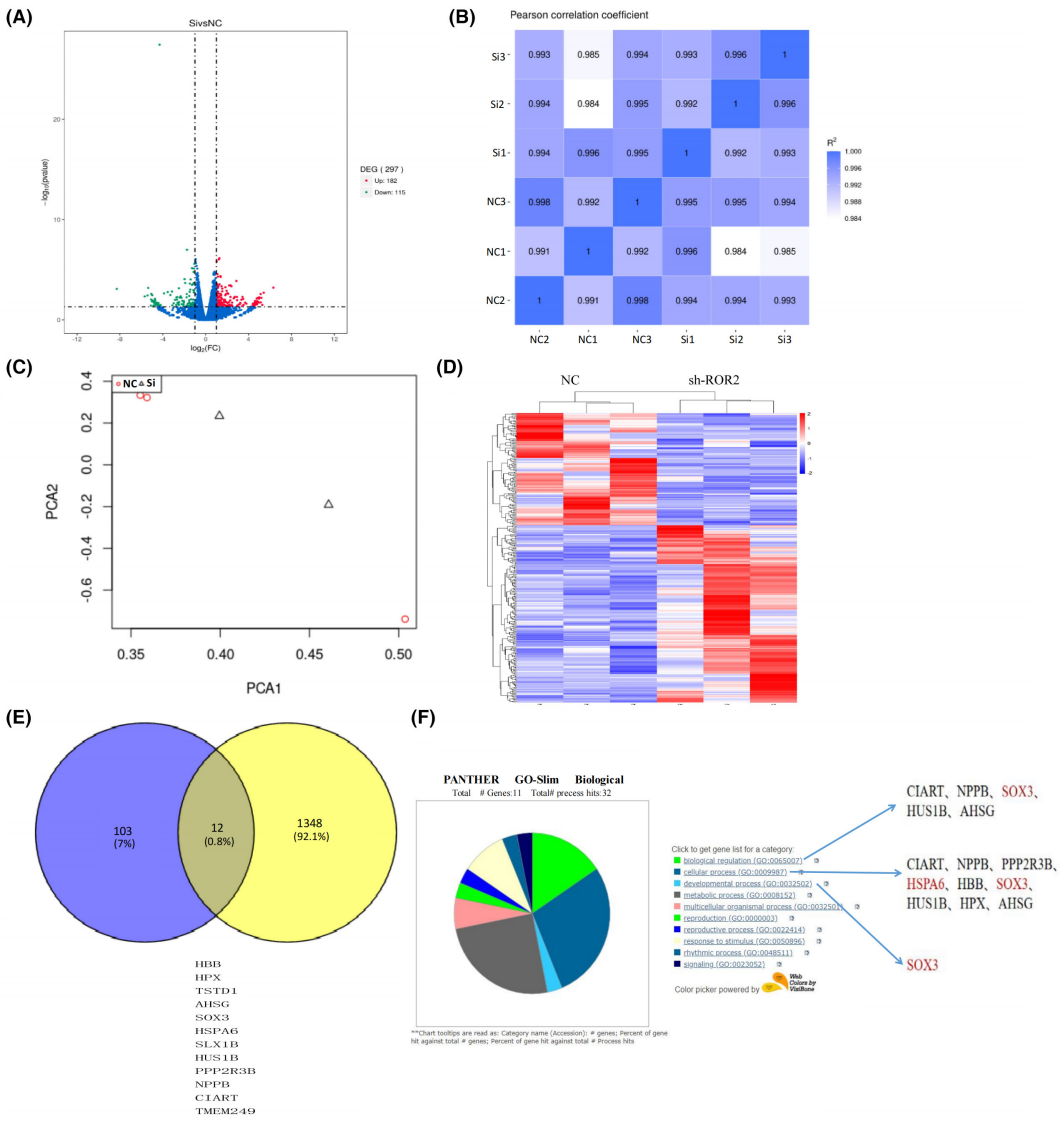
In vitro, genomic expression of the ROR2 in human derivative AC16 cells. (A) A volcano plot analysis represents the magnitude of the effect of the genomic expression on the AC16 cell line. (B) Represents the principal component analyses of the gene expression of the AC16 cell line. (C) To evaluate the differences between groups and the repeatability of samples within groups, red is the normal group, and blue is the *ROR2* knockdown group. (D) Represents the heat map shows high expression in red and low expression in blue. (E) The down‐regulated genes in the cell transcriptomic sequence intersected with down‐regulated genes in the tissue transcriptomic sequence. (F) GO ontology analysis of 12 differential genes showed that they were mainly enriched in biological regulation, cellular process and developmental process.

We further performed the complete transcriptome analysis on AC16 cells, including control (negative control siRNA‐transfected) and transfected with siRNA to knock down the *ROR2*. The same screening criteria further reveal the 297 genes altered in the ROR2‐KD group, of which 182 are upregulated and 115 genes are down‐regulated (Table S2), as shown in the volcano plot (Figure 4A). The heat map correlation matrix was also drawn to visually display Pearson's correlation coefficient of gene expression, indicating the higher similarity of expression patterns between samples (Figure 4B). In addition, the principal component analyses indicated the separate clustering of both groups, suggesting that each group's gene expression members are highly correlated (Figure 4C). Likewise, the heat map indicated pronounced transcriptome alterations in the ROR2‐KD group compared to the control group. The heat map shows high expression in red and low expression in blue (Figure 4D). We intermingled the transcriptome sequencing results of cells with the transcriptome sequencing results of tissues with genes that were down‐regulated for ROR2. The intersection of down‐regulated genes was taken found in a total of 12 common HBB, HPX, TSTD1, AHSG, SOX3, HSPA6, SLX1B, HUS1B, PPP2R3B, NPPB, CIART and TMEM249 (Figure 4E). GO ontology analysis was performed on 12 ROR2 co‐down‐regulated genes. The results showed that the 12 genes were mainly enriched in three biological processes: biological regulation, cellular process and developmental process (Figure 4F). So we chose SOX3 and HSPA6 as the follow‐up research targets.

### 3.5 In vivo, AAV9 was used to KD the *ROR2* in the SD rat

**FIGURE 5:**
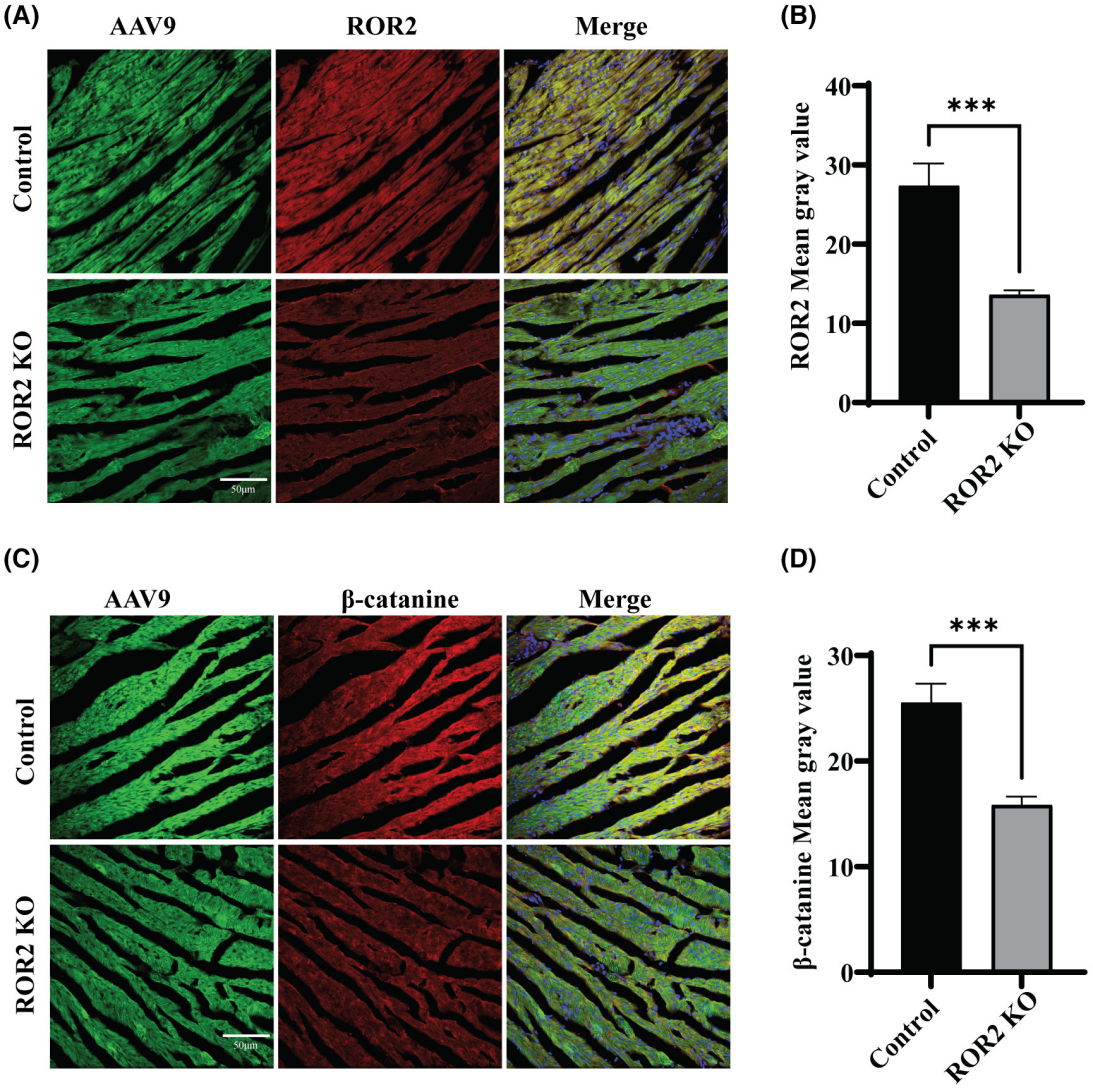
In vivo, AAV9‐ROR2‐RNAi (ROR2‐KD) and AAV9CON534 (control) were used to establish the SD rat modal. (A) Represents the immunofluorescence images of ROR2 in the heart of SD rats. (B) Represents the quantitative analysis of the ROR2 intensity level in the Rats. (C) Represents the β‐catenin intensity level in the heart tissue sample of the Rats. (D) Represents the quantitative analysis of the β‐catenin intensity level in the Rats.

**FIGURE 6:**
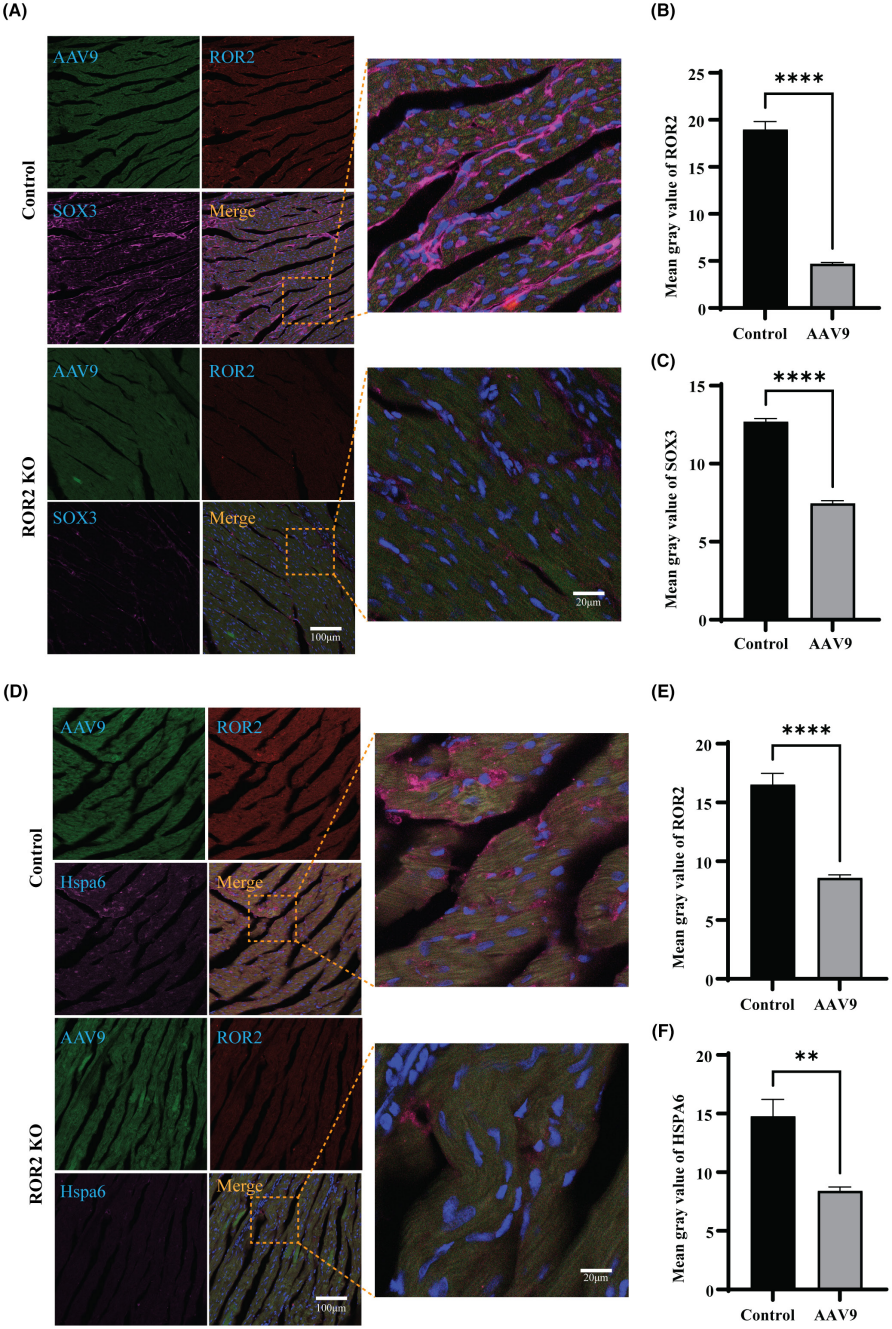
In vitro, the HSPA6 was down‐regulated in response to KD ROR2 via β‐catenin and SOX3. (A) Represents the immunofluorescence images of ROR2 and SOX3 in the heart of SD rats. (B) Represents the quantitative analysis of the ROR2 intensity level in the Rats. (C) Represents the quantitative analysis of the SOX3 intensity level in the Rats. (D) Represents the ROR2 and HSPA6 intensity levels in the heart tissue sample of the Rats. (E) Represents the quantitative analysis of the ROR2 intensity level in the Rats. (F) Represents the quantitative analysis of the HSPA6 intensity level in the Rats.

Furthermore, to study the *ROR2* downstream signalling in vivo, the animal model was established by knocking down the *ROR2* using AAV9. In order to verify the successful construction of ROR2 knockdown SD rat model, the following experiments were performed. Immunofluorescence was used to detect the expression of GFP and ROR2 protein in the myocardial tissue of the normal group and the knockdown group. Compared with the control group, the expression of ROR2 in the myocardial tissue of the knockdown group was significantly reduced (Figure S2A). The expression level of ROR2 protein in the corresponding tissues was detected by western blot. Compared with the control group, the protein expression level of ROR2 in the knockdown group was significantly decreased, further confirming the rat model's successful construction (Figure S2B, *p* < 0.01). B‐ultrasound was used to take pictures of the hearts of the model rats, as shown in Fig.: Compared with the control group, ROR2 gene knockdown rats had significantly increased right ventricle hypertrophy. The hearts of the two groups of experimental rats were removed to measure the radius and weight (Figure S2C,D, *p* < 0.05). The results showed that the weight and volume of the ROR2 knockdown heart were significantly larger than those of the control group, further confirming the potential significance of ROR2 in TOF disease. The heart tissue of the rat model was incubated with the ROR2 antibodies. The immunofluorescence images show a significantly low expression of ROR2 in the ROR2‐KD group compared to the control group (Figure 5A,B, *p* < 0.001). Moreover, the heart tissue was further incubated with β‐catenin, and the results indicated high expression in the control group compared to the ROR2‐KD group (Figure 5C,D, *p* < 0.001). The heart samples of rats incubated with SOX3 and HSPA6, and ROR2 to further describe the downstream signalling. IF images of samples reveal a significant decrease in SOX3 expression in ROR2‐KD compared to the control group (Figure 6A–C, *p* < 0.0001). Similarly, the results indicated that HSPA6 expression was decreased in the ROR2‐KD group than in the control (Figure 6D–F, *p* < 0.001). These results suggest that the ROR2 regulates the HSPA6 expression.

### 3.6 In vivo and in vitro, the ROR2 regulates the HSPA6 via β‐catenin and SOX3

**FIGURE 7:**
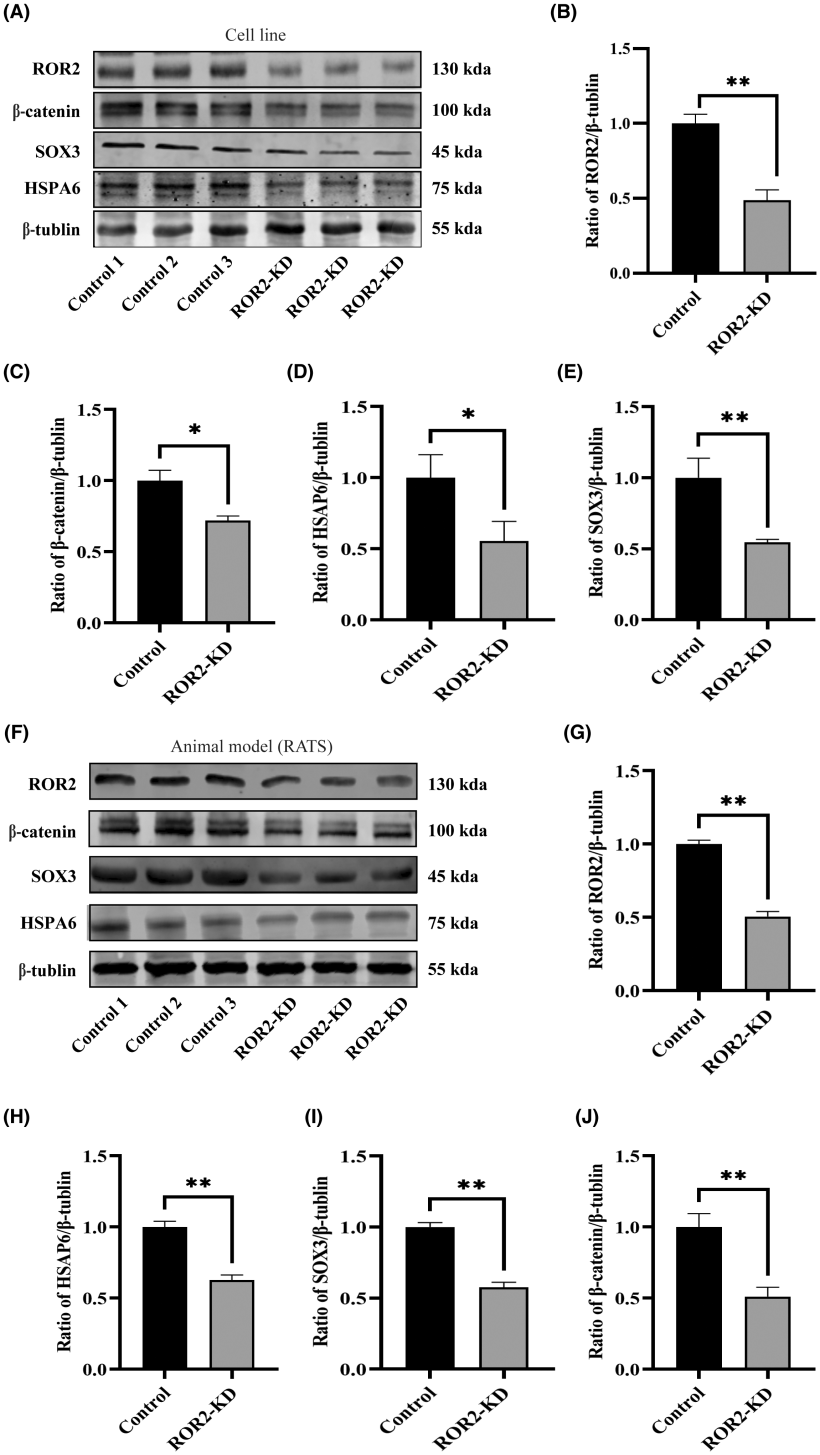
In vitro and in vivo, western blot analysis indicates the ROR2 affects the HSPA6 via β‐catenin and SOX3. (A) Represents the gel images of western blot have been displayed ROR2, β‐catenin, SOX3 and HSPA6 in the AC16 cell line. (B) Represents the quantitative analysis of the ROR2 in the AC16 cell line. (C) Representing the quantitative analysis of the β‐catenin in the AC16 cell line. (D) Represents the quantitative analysis of the SOX3 in the AC16 cell line. (E) Represents the quantitative analysis of the HSPA6 in the AC16 cell line. (F) Represents the gel images of western blot have been displayed ROR2, β‐catenin, SOX3 and HSPA6 in SD rats. (G) Represents the quantitative analysis of the ROR2 in the SD rats. (H) Represents the quantitative analysis of the HSPA6 in the SD rats. (I) Represents the quantitative analysis of SOX3 in the SD rats. (J) Represents the quantitative analysis of the β‐catenin in the SD rats.

To further confirm the hypothesis that ROR2 regulates the HSPA6 via β‐catenin and SOX3 in vivo and in vitro, we performed the western blot analysis in rat models and AC16 cells. In vitro, the gel images of western blot have been displayed (Figure 7A). The analysis of western blot gels demonstrated the ratio of ROR2 protein expression in the ROR2‐KD group was significantly decreased compared to the control group (Figure 7B, *p* < 0.001). Additionally, the results show that the protein ratio of β‐catenin, SOX3 and HSPA6 was significantly decreased in the ROR2‐KD group corresponding to the control group (Figure 7C–E, *p* < 0.05). Similarly, in vivo, the gel images of western blot were displayed (Figure 7F). The western blot analysis showed low protein ROR2 expression in the ROR2‐KD group (SD rats) compared to the control group (Figure 7G, *p* < 0.001). Moreover, the quantification of western blot shows that the protein ratio of β‐catenin, SOX3 and HSPA6 was significantly decreased in the ROR2‐KD group (SD rats) corresponding to the control group (Figure 7H–J, *p* < 0.001), and the results were parallel with in vitro. To confirm that ROR2 regulates HSPA6 expression through β‐catenin signalling pathway, a plasmid overexpressing ROR2 was transfected into human cardiomyocytes AC16. western blot results showed that ROR2 protein expression was significantly increased after AC16 cells were infected with the overexpressed virus (Figure S1F, *p* < 0.01). Overexpression of ROR2 also increased HSPA6 protein expression compared with control (Figure S1F, *p* < 0.05). AC16 cells overexpressing ROR2 were added with a β‐catenin signalling pathway inhibitor. The addition of the inhibitor MSAB (Mce) significantly reduced HSPA6 protein expression compared with control cells, which was consistent with the effect of ROR2 knockdown (Figure S1G, *p* < 0.01). This strengthens our belief that ROR2 regulates HSPA6 expression through the β‐catenin/SOX3 signalling pathway, providing some clinical implications for treating TOF in congenital heart disease.

## 4 DISCUSSION

Tetralogy of Fallot is one of the most common congenital heart diseases developed during embryonic development. In embryonic development, successful cell‐to‐cell coordination is required to provide appropriate direction, resulting in cell proliferation, differentiation and apoptosis.^9^ Right ventricular hypertrophy is a secondary process resulting from the hemodynamic overload proper of the malformation. This study aimed to investigate the specific mechanisms by which *ROR2* deletion or downregulation may indirectly lead to right ventricular enlargement in TOF‐related symptoms. Immunohistochemical analysis of human heart tissue samples showed that the expression of ROR2 was significantly decreased in the TOF patients compared to the control group. Furthermore, western blot and RT‐qPCR results showed that the ROR2 level was significantly higher in the control compared to the TOF. These results propose that ROR2 expression is down‐regulated in TOF. In support of this study, previous researchers found that ventricular septal defects were found to be associated with ROR2‐deficient mice.^14^ As research has progressed over the past decades, several studies have shown that regulatory molecules such as transcription factors, receptors and growth hormones play essential roles in heart development. However, their role is unspecific and not limited to septum formation.^25^, ^26^, ^27^, ^28^ In addition, other studies have shown that ROR2 is essential for maintaining cardiomyocyte proliferation.^9^, ^29^ Besides that, ROR2 appears to play a dual role as a tumour suppressor or activator in certain types of cancer.^30^ Additionally, it has been reported in humans that ROR2 mutation is responsible for Robinow syndrome and congenital heart disease in around 15% of the patients.^31^

Furthermore, potential interactions between gene expression and TOF were identified in this study. Interestingly, this effect was even more pronounced, with 2246 genetic alterations reported in TOF, of which 886 were upregulated and 1360 were down‐regulated. ROR2, SOX3 and HSPA6 were among the top 17 down‐regulated genes associated with TOF patients. Similarly, in vitro, the whole genomic analysis was performed on AC16 cells, including control (non‐transfected) and transfected cell line (ROR2‐KD). The mRNA expression in the ROR2‐KD group was parallel to TOF samples. Additionally, heat maps showed a significant transcriptome alteration in the ROR2‐KD group.

In vitro experiments confirmed that interference with ROR2 promoted apoptosis and curtailed cell proliferation. The transfected AC16 cell displayed low expression of the Edu and migration activity, suggesting the growth rate of cells progressively declined. Likewise, previous publications explore the role of ROR2 in cell lines, and they indicated that knockdown of the ROR2 suppresses proliferation and promotes apoptosis.^13^, ^32^ Furthermore, Wright et al. reported that Knockdown ROR2 is involved with the downregulation of matrix metalloprotease‐2, suppressing cell migration and growth in soft agar.^33^, ^34^ A flow cytometry analysis indicated that the percentage of the cell cycle in the S phase was elevated in the ROR2‐KD group; nevertheless, the G2/M cell cycle stagnated, and the apoptosis of ROR‐KD cells was significantly increased. Similarly, Castro et al.^13^ also reported that ROR2 slowed the G1 to S and G2 to M phase transition.

This study further reports that ROR2 regulates the HSPA6 via β‐catenin and SOX3 in vivo and in vitro. In vitro studies showed that the proportion of β‐catenin, SOX3 and HSPA6 proteins was decreased in the ROR2‐KD group. Henry et al.^15^ reported that ROR2 might regulate both β‐catenin‐dependent and β‐catenin‐independent Wnt signalling pathways. Previous studies have shown that SOX3 is critical in interacting with β‐catenin and regulating β‐catenin‐dependent pathways during embryonic heart development.^18^, ^35^ In this study, in vivo, the animal model was established by knocking down the ROR2 using AAV9. Similarly, in vivo, the proportion of β‐catenin, SOX3 and HSPA6 proteins declined in the ROR2‐KD group (SD rats), and the results were parallel with in vitro. Previously, HSPA6 protein was found to be associated with apoptotic response and cell differentiation. The primary function of HSPA6 is to protect differentiated human neuronal cells from cellular stress.^36^ In addition, during human embryonic development, the apoptotic pathway and cell cycle are regulated by HSPA6, the protective role of embryonic cells from cellular stress, including chemical teratogens.^36^ Therefore, these results indicate that ROR2 regulates the downstream signalling pathway of HSPA6 through β‐catenin and SOX3.

In conclusion, this study provides evidence of impaired ROR2 function in TOF patients. Moreover, in vivo experiments demonstrated that ROR2 exerts a suppressive effect on apoptosis and promotes cell proliferation. Additionally, the study revealed an increase in the percentage of cells in the S phase and a blockage of the G2/M cell phase. In vitro experiments using the SD rat model indicated that ROR2 regulates the expression of HSPA6 through the β‐catenin/SOX3 pathway. Western blot results also confirmed the downregulation of HSPA6 protein expression via β‐catenin/SOX3 following ROR2 knockdown in both in vivo and in vitro settings. These results suggest that ROR2 plays a critical role in the development of TOF by inhibiting cell proliferation and promoting apoptosis, which is mediated by the downregulation of HSPA6 through the β‐catenin/SOX3 signalling pathway.

## AUTHOR CONTRIBUTIONS

**Rui Guo:** Conceptualization (equal); methodology (equal); writing – original draft (equal); writing – review and editing (equal). **Yiran Zhou Guo:** Writing – review and editing (equal). **Qing Zhou:** Writing – review and editing (equal). **Guoju Li:** Formal analysis (equal). **Zhanghui Du:** Formal analysis (equal). **Yefei Shi:** Formal analysis (equal). **Quansheng Xing:** Funding acquisition (lead); resources (equal); supervision (equal); writing – original draft (equal); writing – review and editing (equal).

## FUNDING INFORMATION

This work was supported by grants from The National Natural Science Foundation of China (NSFC) (Grant No: 8170315 and 82071583) and the Taishan Scholarship Program.

## CONFLICT OF INTEREST STATEMENT

The authors declared no conflict of interest.

## Supporting information


Figures S1–S2:

Table S1:

Table S2:

Table S3:

## Data Availability

All data generated or analyzed during this study are included in this article. Further inquiries can be directed to the corresponding author.
