# Supplementary material for: ROR2 deficit may induce the tetralogy of Fallot via down‐regulating of β‐catenin/SOX3/HSPA6 in vitro and in vivo
Source: J Cell Mol Med. 2023 Sep 25;27(22):3539–52. doi: 10.1111/jcmm.17969 (PMC10660643; doi:10.1111/jcmm.17969)
Supplement: Supplementary file 1 — Figures S1–S2: [file JCMM-27-3539-s001.zip › suplymentery figure 1-2.docx]

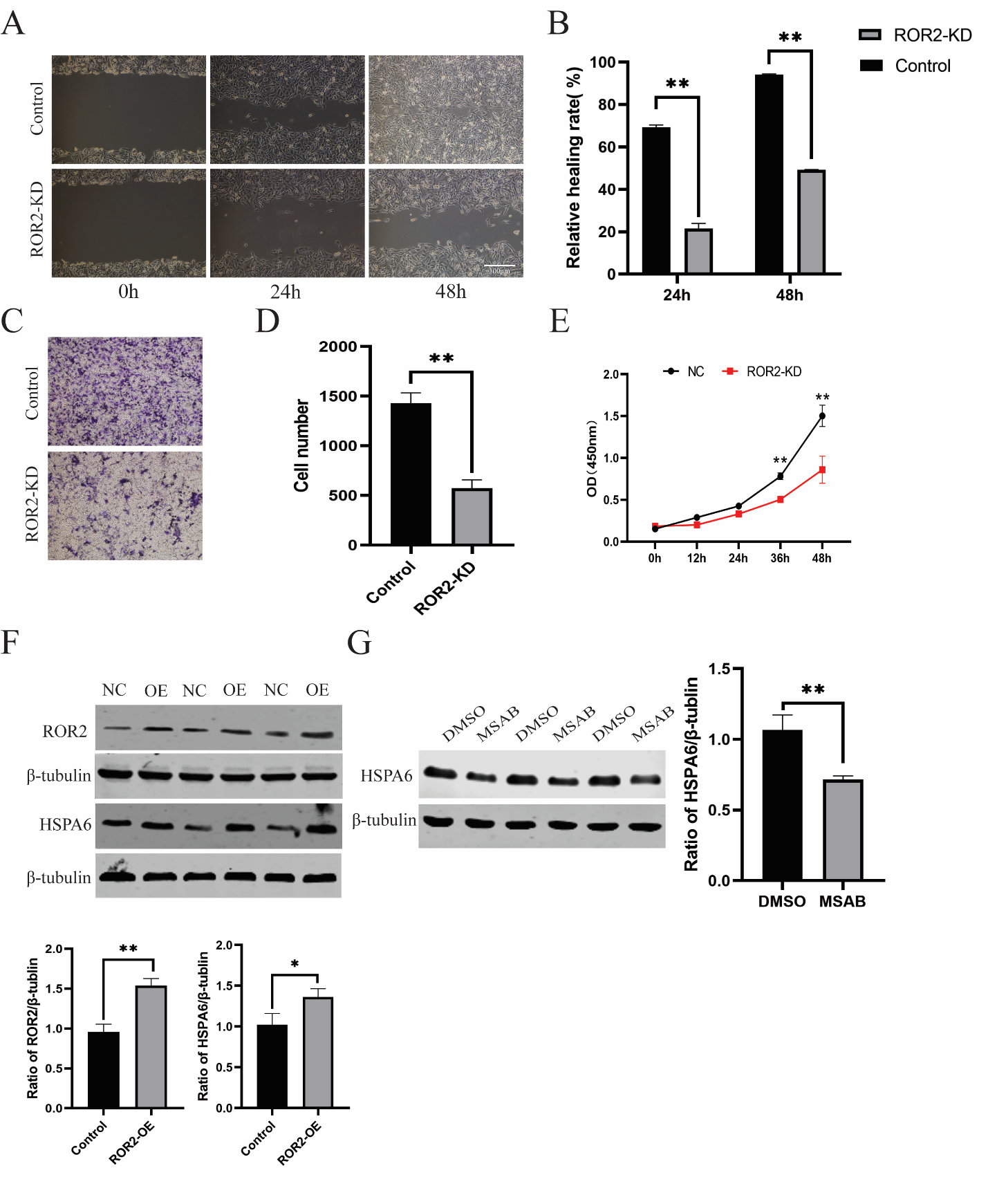


**Supplementary Fig. 1:** **Knockdown *ROR2* in the AC16 cell line reveals the growth rate of cells gradually decreased. (A)** Represents the cell migration activity of cells at 24h and 48h after cutting the cells cluster. **(B)** Represents the cell migration activity analysis between the *ROR2*-KD cell and control group. **(C)** Represents the transwell chambers experiments, the cell was stained with crystal violet. **(D)** Represent the quantitative analysis of the transwell chamber experiment. **(E)** Represents the CCK-8 cell viability assay evaluating the cell proliferation between the control group and *ROR2*-KD group at 12h, 24h, 36h, 48h, and 72h. **(F)** Western blot bands and statistical plots of ROR2 protein and HSPA6 protein after overexpression of ROR2. **(G)** Protein bands and statistical plots of HSPA6 protein detected by western blot after overexpression of ROR2 and addition of inhibitor MSAB.

**
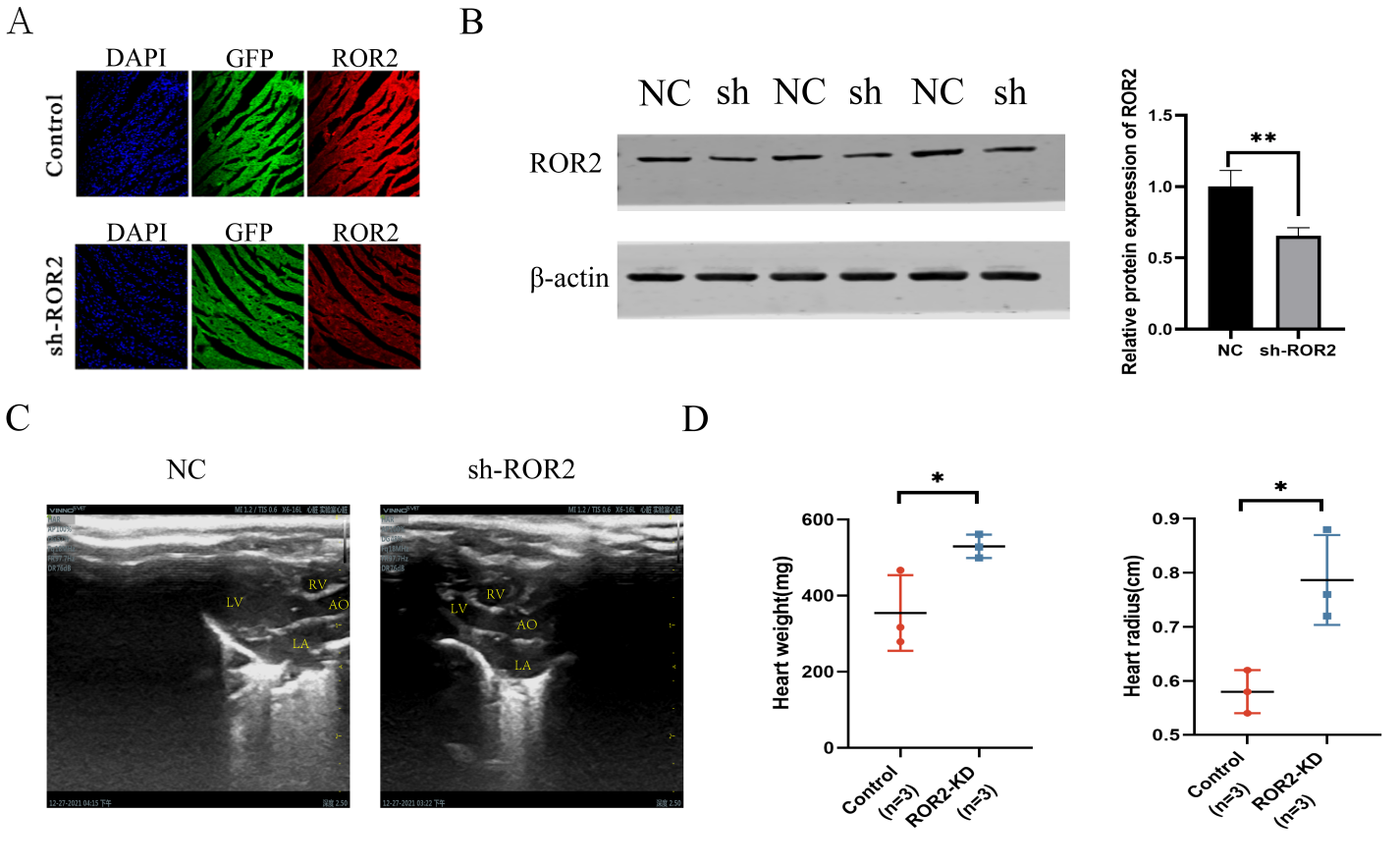
**

**Supplementary Fig. 2:** **The *ROR2* gene knockdown SD rats were successfully constructed.** (A) ROR2 protein expression in SD rat myocardial tissue was detected by immunofluorescence. (B) The expression of ROR2 protein in rat myocardial tissue was detected by western blot to verify whether the knockdown model was successfully constructed. (C) The enlargement of the right ventricle in the hearts of the knockdown group and the control group was detected by B-ultrasound; RV marker indicates the position of the right ventricle of the rat heart, LV marker indicates the position of the left ventricle of the rat heart, LA marker indicates the position of the left atrium of the rat heart, and AO marker indicates the position of the aorta of the rat heart.(D) measurement of rat heart weight and radius.
