# Supplementary figures and images for: ROR2 deficit may induce the tetralogy of Fallot via down‐regulating of β‐catenin/SOX3/HSPA6 in vitro and in vivo
Source: J Cell Mol Med. 2023 Sep 25;27(22):3539–52. doi: 10.1111/jcmm.17969 (PMC10660643; doi:10.1111/jcmm.17969)

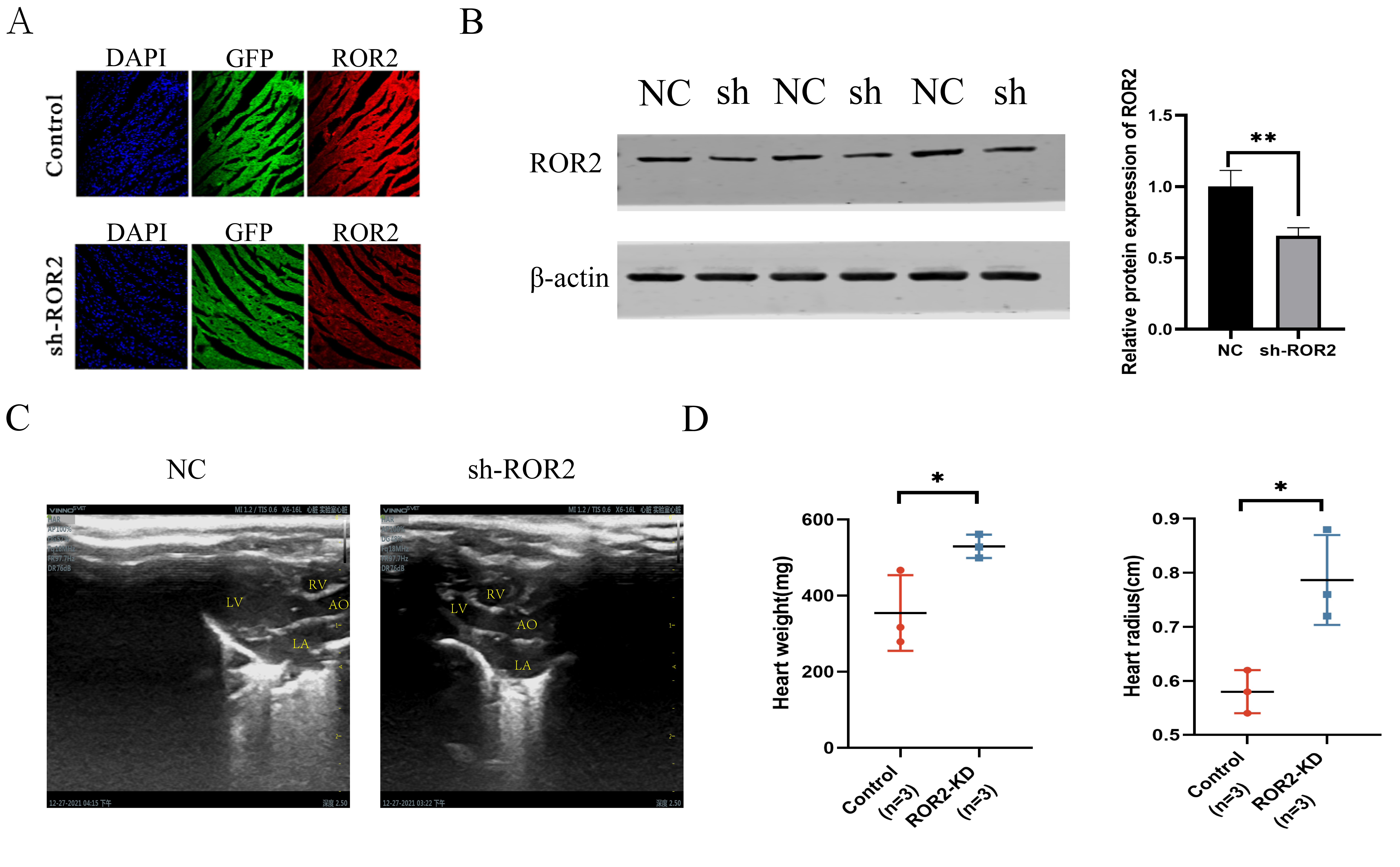

Supplement: Supplementary file 1 — Figures S1–S2: [file JCMM-27-3539-s001.zip › suplymentery figure 2.tif]

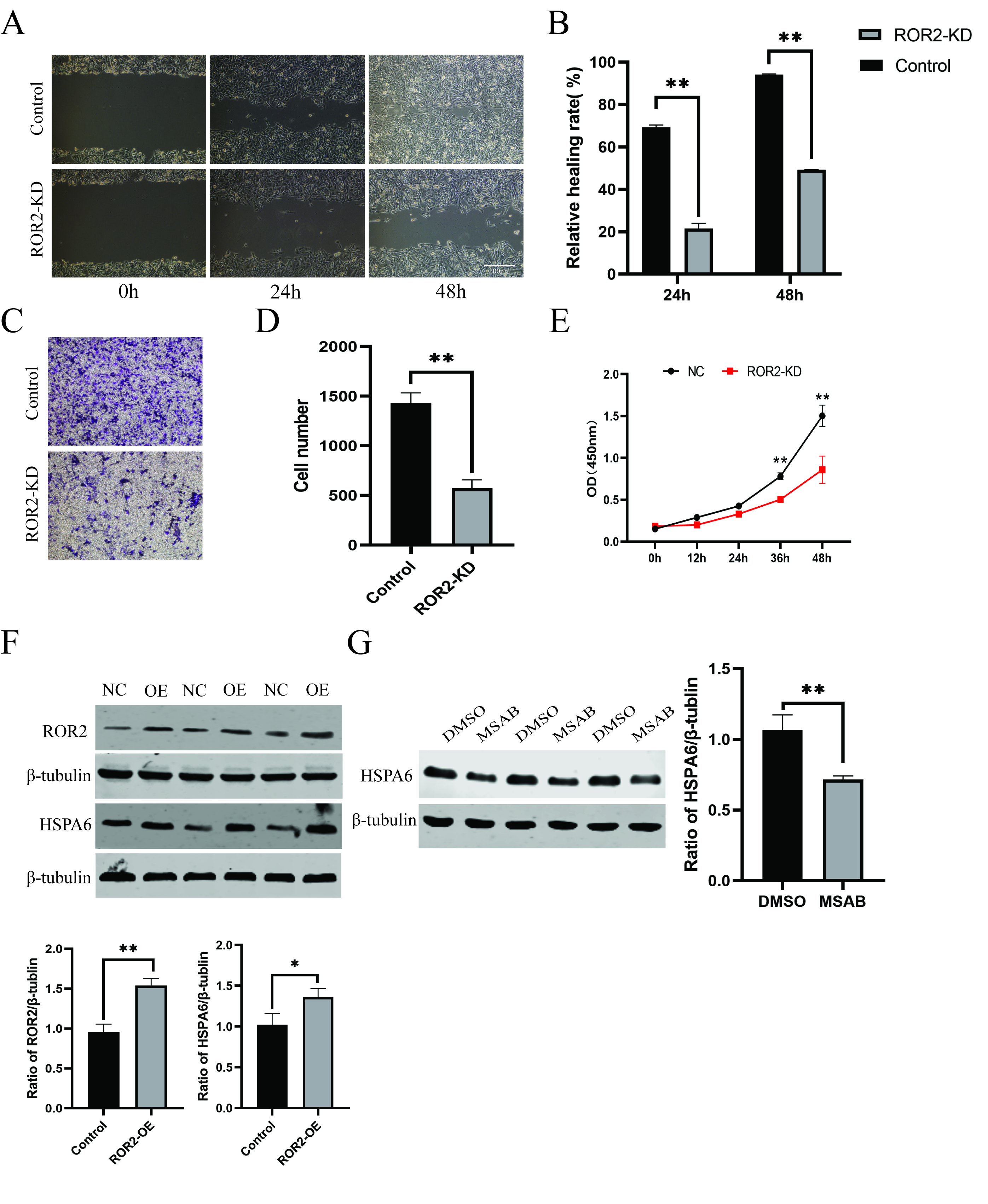

Supplement: Supplementary file 1 — Figures S1–S2: [file JCMM-27-3539-s001.zip › suplymentery 1.tif]
